# Sabbath Clinical Benevolence

**Published:** 1860-08

**Authors:** Alex. Means

**Affiliations:** Oxford, Georgia


### ARTICLE II.

### *Sabbath Clinical Benevolence;* By ALEX. MEANS, M. D., Oxford, Georgia.

*Messrs. Editors:* Although I profess to be a sincere admirer of that heavenly philanthropy which melts the heart and opens the hand to the relief of human suffering, there is in these modern days a specious kind of *clinical* benevolence, whose claims to my admiration I think not entirely unquestionable.

When Disease, in the course of its indiscriminate depredations upon the health and happiness of our race, has settled upon its victim in a village or neighborhood, and the emaciated frame, pallid cheek and parched lip of a suffering fellow-creature plead feelingly for the hand of relief, that unaffected charity which is quick to hear, and swift to obey the call of distress, claims and receives my heart's highest tribute of admiration, and especially when I recognize it in man's amiable and sympathizing *counterpart.*

When female loveliness, regardless of the studied fripperies of dress, and the meaningless formalities of fashion, and moved by those kindly and benevolent emotions which characterize the *heart* and distinguish and enoble the *soul* of woman, is seen pressing to the house of sickness, and bending over the bed-side of disease, unostentatiously administering by a thousand kind offices, to the comfort and relief of the stricken and languishing invalid, then does beauty wear a diamond lustre, and exert an undefinable but captivating power over the yielding soul of our sex—winning our profound respect, and enkindling a deep and virtuous affection, which the butterfly decorations of dress, or the ephemeral blandishments of the ball-room can never—*never* inspire.

But during many years' intercourse with the afflicted, I have frequently been called to witness sick-bed scenes, when the circumstances forbade me to hope that the short-lived, but often extravagant attentions bestowed by occasional visitors, proceeded from the warm region of the heart, where true philanthropy resides. Such visitors come, it is true, to visit the sick, and especially on that "first best day of all the seven," on which the laws of the land and an enlightened public opinion, exact a temporary respite from their secular employment. Nor does the evil which provokes the present reluctant stricture, confine itself exclusively to any condition of civilized society, or to any class or caste in community.—Its traces are seen in our populous towns and steepled cities, while our retired villages and thriving country places, give frequent and patent exhibitions of its prevalence, which cannot have escaped the unwelcome notice of every discerning physician. On the "Day of Rest," these, after a late morning's meal and a protracted toilet have prepared them to undertake the task, and meet the public eye, they set out upon their mission of love (?). Or, it may be, after the tedious sanctuary service has closed, and the weary worshippers have baited hugely, and snoozed snugly for an hour, as if to indemnify their over-taxed and panting patience, that they rise in readiness to exercise charity to the woes of the world, and start out with commendable self-sacrifice and re-invigorated purpose, to endure the monotonous scenes of the lingering day, or while away the irksome hours of its remaining sun-light. There are seen men of high and low degree—the "gentle" and the "simple," all clad in their Sunday's wardrobe, and making their way to the home of suffering—some freshly shorn on cheek and chin—others with countenances profusely draped with Aaronic honors, and now and then a precocious youngling, nursing an unfledged mustache, tender as the down upon a duckling's back; while whole bevies of the garnished fair, swimming in crinolines and calicos, silks and muslins, and accompanied by some half dozen restless and rollicking inmates of the nursery, unite in the cavalcade which moves towards the chamber of the sick. They reach the place of destination. It is, perhaps, a quiet and retired cottage, surrounded by the implements and scenery of rural life. The ladies enter with noiseless footsteps, and are gently welcomed by some of the household. In the meanwhile, the doe-skins and danglers who have officiously performed the important duty of gallants, snugly esconce themselves, with curved spine and well-braced heel-taps, upon the topmost rail of the neighboring fence, to puff pig-tails and whittle sticks by the hour, until the waning day brings out the softer sex, to smile them from their perch and reward them for their patience by the soft dalliance of an evening's walk.

The visitors, once entered, flock into the sick-room where a mother or daughter lies in anguish—occupy every corner, and hang over the bed-side of the oppressed patient, until, from the want of free ventilation in her crowded apartment, the romping and crying of the petulent "small-fry," and the stale, oft-repeated, and teazing interrogations of, "How do you feel to-day?" "Did you rest well last night?" "What shall I do for you?" "Do take some soup—try a little butter-milk," &c., &c., the panting sufferer and her impatient physician, would most gladly have transferred a *portion* of this glut of Sabbath kindness, to the lonesome hours of the approaching night and subsequent week. But, alas! this desire, though often intimated, and sometimes half expressed, is unheeded and unobtained. So soon, however, as the Sabbath sun sinks low in the western skies, and lengthening shadows begin to steal across the plain, there is generally a bustle in the apartment of the sick. The sympathizing visitors rise to go. One, and another, and another, is respectively entreated, by some anxious friend or relative, to remain through the night, and alternate with those already worn out with watching and fatigue, in administering to the wants of the languishing patient. The kind appeal, however, is commonly met by some apologetic recital of coughs or colds, or recent accidents, or some pressing domestic duties just recollected, which, most unfortunately, prevent their attendance for the night; but they *all*, yes, every creature of them, "will call again shortly." In sooth, the proverbial adroitness of "College boys" in supplying excuses for absence from morning prayers, finds many a counterpart in older circles.

But no more can now be urged. They all depart to their respective homes and fire-sides. The yard fence, too, that enclosed the lonely cabin, is now deserted. The lounging idlers of *our* sex, that had peopled its crazy rails, have left it once more to the ominous chirp of the cricket, or the stealthy tread of the scared green lizard returning to its haunts.—Gradually the subdued sun-light melts away in the calm, blue West, and darkness drops her curtain upon the world, while the sleepless child of affliction, tossing upon her fever-heated couch through the long and dreary hours of night, is indebted alone to the benevolent vigilance of one or two humane neighbors, in aid of her almost exhausted family, for those necessary services and nameless little attentions which soothe the excited mind, and rob disease of much of its poignancy.

But night passes away. The powerful "King of Day" again smiles from the eastern heavens, and mounts in grandeur up their broad, blue arch, as if to cheer, by the very exuberance of her beams, the lonesome chamber where the sufferer lies. He stays not in his course, however, until his bright, diurnal career closes in the glowing occident, while the pale but pitying stars seem silently to emerge from their invisible homes in the deeps of ether, to shed their soft and lambent light over the deserted earth and skies, and whisper of hope and Heaven.

For six successive periods have the vicissitudes of day and night passed upon the world, while few and seldom have been the visits to the house of disease, until the still, dozy hours of another Sabbath, agains stirs the hebdomadal benevolence of some sympathizing neighbors, and the sick-room is again thronged with an inquisitive and officious crowd.—On occasions like these, a scene such as we have attempted to portray in the dramatic representation following, must have presented itself, in whole or in part, to the reluctant observation of almost every physician who has claims to experience in the practice of his responsible and benevolent profession.

SCENE—(A sick-room—small and imperfectly ventilated.)

*DRAMATIS PERSONÆ.*

MRS. AILING, (the patient). MR. AILING, (her husband).—MRS. GOODLOVE, (a pious and attentive friend). Physician and Nurse.

MR. AILING—(Looking out at the only window). My dear wife—a trial is approaching for you. Yonder comes your Sabbath visitants—their number largely replenished by children and dogs. I dread your interview with the idle, garrulous group.

PHYSICIAN—Mrs. Ailing, conversation will exhaust you, and may superinduce a relapse, from which it may be difficult to recover. You must refrain, therefore—keep quiet and cool, and let your talkative friends deal on their *own* capital. Alas! it is to be regretted that their stock in trade is not likely soon to fail.

(Enter MRS. OVERMUCH, MRS. WISEACRE, and MRS. ALARM, with a half dozen children. All go directly to the bed-side).

MRS. OVERMUCH—Why, raly, I'm sorry to see you so low, Missez Ailin'; I never know'd so much as that ye were complainin' untel last week. How do ye feel to-day? What hurts ye? Do you think ye are any better?

PATIENT—(With parched lips and burning brow). Very sick. The Doctor—must tell—it exhausts—me—to talk.

MRS. OVERMUCH.—(Peeping down at her through her glasses,) Well, well! I say it. How ye have fallen off since I seed ye. I would hardly a' knowed ye. Where is yer pain? I do wonder ef you're got the lung fever?

PHYSICIAN.—Suffer me to reply, madam, as Mrs. Ailing's great debility renders it extremely imprudent to attempt conversation. Symptoms seem somewhat more favorable this morning, and with proper regard to diet, regimen, &c., a speedy convalescence may be reasonably expected.

MRS. ALARM.—(In a loud whisper to Mrs. Wiseacre, after they have taken their seats,) La, me! did you ever see such a 'natomy! Her eyes look like they were death-struck now.

I wonder if her nails aint turnin' blue? I don't think she'll live till mornin'. She looks for all the world like my poor neighbor, Mrs. Mortal, who died t'other day.

MRS. WISEACRE.—(With a knowing wink,) Ah! I've seed a heap of sickness in my time. "Ye can't ketch an old bird wi' chaff." She's in a slim way, I tell ye. She's been phys-ic'd a'most to death, I'll venter. Suse Trollop told me as how she has tuck, fust and last, fully a'most half a teaspoon-ful o' Calomy! (lifting up both hands in horror.) You can't fool me wi' Calomy—the poison truck! Jim Needless tuck a dost ten years ago, and he's had a pain in his knee afore every wet spell, since last hog-killin' time, and the steam doctor said it was the Calomy. So he tuck him through a course of Lobely, and give him a corn-sweat, and swellered him up wi' bilin' hot blankets, afore ever he could get the canker out o' the cretur's bones.

MRS. ALARM.—Dear me, alive! It' a massy some of my childer hav'n't been killed by it aready; for the doctor has give it to 'em lots o' times, when they've had the fever, but surprizin' to say' they've gener'ly got well, arter a dose or two. (Then in a loud whisper, glancing at the patient)—Look how she breathes! (grasping her arm, half way between the wrist and elbow, like seizing a poker,) As I'm a livin', she hasn't got a bit o' pulse! Dear me, if Dr. Dozy had been here, I'll bet my old gander he'd a had her up afore this. He's my doctor.

MRS. OVERMUCH.—So I say; I wonder they haven't had somebody else here. Do ye think we could prevail on 'em to send for Dr. Blarney?—he's so obligin' and easy like. He never comes to our diggin's, but what he calls to ax for a drink o' water, or somethin', and allers takes up our little greasy Bets, and smacks lips. Why, let me tell you; last Sunday, arter meetin, he walked home with our Nance, with a whole gingercake in his pocket for Bets. He cotch her comin' from the dinner pot, whar she'd been soppin' the pot licker, tuck her upon his knee, and the way he did buss her was a caution to old bachelors. Bless the man! He knows the valler of daughters.

Mrs. Wiseacre.—Well, you may all think as you please, but it aint my judgment that all the doctors in the univarsal world can save this 'oman. Mrs. Overmuch, you knowd Old Mother Tuggle. She was down for six months, for all the world like Mrs. Ailin'. She used to beg for warter, jist like this 'oman, and her skin was hot, too, jist like her'n, (Winking and nodding significantly.) I reckon I can see as far into a millstone as him that pecks it. And I know the doctor's truck never done her a bit o' good, and he worked hard, too, arter he got thar, and that mout a been a matter of two or three hours afore she died. I tell ye, them are "*slow fevers*" are monstrocious hard to break.

Mrs. Goodlove.—(coming in cautiously from an opposite room, and addressing the patient in accents of tenderness,) Dear Mrs. Ailing, I have had some nice chicken soup made, according to the doctor's directions, which I hope you will relish, and which I know will comfort and nourish you.

Mrs. Ailing.—Thank you, thank you, my kind friend.

Mrs. Overmtch.—Missuz Ailin', won't you have me to do something for you? Your head don't lie right. (Adjusting the pillow.) Can't you eat something that I could make for you? That soup's too wishy-washy. Mayby you'd eat a little dried beef, or some fried crab-lanterns. And now I think of it, I've got some number-one sour-crout at home, and I wouldn't mind givin' you as much as you could eat. Ingurns, too, is a mighty relish, fried *rale done*.

Physician.—Mrs. Ailing does not desire such food, I am sure, ma'am; and if she did, prudence would forbid its use, at present, as it is extremely indigestible, and unwholesome to one in her condition.

Mrs. Overmuch.—Well, but doctor, only a *leetle*, you know; only a few mouffuls at a time wouldn't hurt. I used to give my son Johnny o'most anything he craved, when he had been sick, and was a leetle on the mend; and if it hadn't been for an onaccountable backset he got one day, just arter a hearty bait o' cold leek, hock and fried greens, he would a mended mighty fast. But I ain't a livin' ef he didn't like to kick the bucket that time. Though I never could guess what flung him back.

PHYSICIAN.—I should be by no means puzzled to account for it myself, ma'am, nor could Mrs. Ailing expect a better fate, if she escaped with her life, were she to load her enfeebled stomach with a diet which might tax the digestive powers of a ploughman or a grubber. I hope, however, both myself and Mrs. Ailing duly appreciate your *intended* kindness.

MRS. OVERMUCH.—Yes, for sartain, sure, I only meant it for her good. Well, Doctor, have ye ever gin her any catnip tea, or sweated her over bitter yerbs yet, sich as worm-wood, pine top and Horehound?—That is wonderful healin. Mrs. Jinkins, did it a few months ago. She was guine about, howsomever, when she begun, and arter a week or two she was te-totally well.

PHYSICIAN.—I have not yet thought her at the steaming point, Ma'am; nor would her malady justify such a mode of treatment.

PATIENT.—Please give me a little toast water to moisten my lips. (Here the three ladies all bustle about, each in the other's way, to serve her at once.)

MRS. WISEACRE.—Poor dear creature!—how helpless she is. Here, Mrs. Overmuch, hold up her dear head. Mr. Alarm, run and fetch some good, cool warter to mix with this 'ere toast warter,—its so oudacious hot. (Patient drinks)—Thar now—it takes me to know what sick folks hone for. My old man allers said I gin him cold warter better nor any body else (dropping her head and squinting significantly over her cracked, full moon spectacles). Look'ee here, Mrs. Alarm, (taking her aside) may be I say it that oughten to say it,—but don't breathe it to a liven soul, (in a loud whisper) Ef ye'll believe my racket, ef Dr. Soakum had just a gin Ailin' the doose, (the cold *douche*) two or three days ago, and ha' packed her well, she mought ha' been a cookin' dinner, or a spinnin' by now.

During the above scene, several other groups come in, and each, in its turn, sallies up to the bedside, and in strains of melancholy condolence, salutes the sufferer. One fair hand adjust the patient's cap-strings, another, smooths back her hair, while a third, pats the *already* well arranged bed-clothes.

Just at this lull, in the conversational storm, Mrs. Overmuch's pouting son Jimmy, knocks an apple out of Sammy Alarm's hand, and gets a *bruiser* upon his flattened nose, from the fiery little pugilist, when both urchins clinch, and pull hair and scratch most lustily.

MRS. ALARM.—(Springing between them). Bless my soul, Sammy what do you mean—right here in the crowd too, and around the sick 'oman's bed. Jerusalem!—it'll never do. Why folks 'll think your mamma hadn't teached ye no manners. But I know better, I've been beatin 'em into ye ever since ye were knee high to a duck. Let that little snub-nosed knoty head alone, then, Sammy, but when you kitch him *out*—gin him "Jessie"—the little runt!—

The Sabbath sun now began to sink low in the western heavens. The group become restless, and a general bustle is heard in the sick room.

MRS. OVERMUCH.—(To the patient) Well I raly wish I could do something more for you. But I must go, for my folks have been lookin' for me, I dare say these two hours—but when I get where the sick is, I never knows when to quit.

PATIENT.—(with feebleness) Please stay—if you possibly can, and watch with me this one night.

MRS. OVERMUCH.—My dear child, I be glad to stay but—but—they all specks me at home, and besides, Bets squalls like all natur, when she wakes up and don't find mammy.—More'n all—I promised Mrs. Liddy Pluck to go down to the *Hangin'* with her bright and yearly in the mornin,' and a body must have rest. That old rogue, Ike, ye know, is guine to turn the corner to morrow, 'corden to law, and I wants to see him off.

MRS. WISEACRE.—(Rising to go) Good evenin' — Missez Ailin'—I'm sorry to leave ye in this fix, but I'm trouble wi'

Rhumaty pains when I 'spose myself. Ye're in a critical siteation, I take it, and I hope folks won't sleight ye. I'll be back, tho,' sure fire, in a day or so.

MRS. ALARM.—Well I must be guine, or it will be dark.

PATIENT.—(Pleadingly) Do pray—don't all leave me.

MR. AILING.—Do Mrs. Alarm, consent to remain through the night with us. I see all the other ladies are going, and we shall be left alone.

MRS. ALARM.—Well, I declare, its unlucky that I can't stay; for though I sort o'jubous that this fever's ketchin,' yet I would stay *ang how*, but Luky and Jince,—The peskey little minxes,—have both cotch bad colds, and have a runnin' at the nose, and I'm afraid to leave 'em arter night to themselves, they kick the kiver off so, when one ain't by 'em.—But I hain't gin ye up for good yet Missez Ailin' and ef I hear ye're a livin' I'll drop in agin to see ye.

MRS. GOODLOVE.—Allow me to say, Mr. Ailing, that I came from home, expecting to remain with your wife to night, and render every attenion in my power. I can do so with perfect convenience, and it will afford me sincere pleasure to serve her. Her condition is slowly improving, and I hope soon to see her and yourself at our humble home.

MR. AILING.—(With a grateful look) Many, many thanks, my dear madam. Your kindness will never be forgotten, and yet you have a higher and noble reward than myself or my loved wife can ever render you, from your own approving conscience, and your smiling God.

Thus all, save one or two tried and truly benevolent frinds, soon pave the way for an exit, and vanished from the room—leaving the philosopher and the christian to deplore the selfishness and insincerity of too many of our race, and contrast that loud boisterous noon-day philanthropy, which submits to no inconvenience, renders no sacrifice and magnifies every trivial service, with that silent, unostentatious benevolence which cheerfully encounters difficulties, and overleaps obstacles, in the order of its zeal to promote the well-being of the suffering and destitute. Such a desinterested, heavenly charity,—in the Bible application of the term,—neither covets popular applause, nor shreaks from the wail of suffering humanity, but shines with bright and steady luster around the midnight couch of disease, as well in the black and fireless hut of poverty as in the glowing and gorgeous chambers of wealth; and in the profusion of its unlimited goodness, delights in acts of mercy and brotherly kindness, as much amid the cares of the busy *week-day* as during the calm of the sacred SABBATH.

